# 

**Published:** 1888-01-14

**Authors:** 


### "THE HOSPITAL" ALMANAC FOR 1888.

This Almanac, published with THE HOSPITAL of January 7th, 1888, was compiled by F. C. Carr-Gomm, Esq., Chairman of the London Hospital, and contains full particulars of the Metropolitan Hospitals in the year of Jubilee, classified as "Endowed," "Chest," "Children's," "Lying-in," "Women's," and "Other Special Hospitals." The results of the collections on Hospital Sunday and Saturday last year, with the cost of raising the former fund; the number of Institutions benefited, and the dates on which these collections will be held in 1888, are given with full particulars of each hospital.

The **TIMES** says: "This Almanac would no doubt be found of great value if suspended in workshops, police stations, and Board schools throughout the metropolis, as well as at the hospitals, where it is likely to find a place, not only in the wards, but also in the out-patient departments."

The **GLOBE** says: "Almost anything one may want to know about any of these institutions may be found there at a glance."

Copies of the Almanac (which is copyright and has been entered at Stationers' Hall) may be had separately on application to the Publisher, 38, Old Jewry, E.C., at the following rates: *If printed on stiff paper*, 6*d*. per copy, or 5*s*. per dozen; if *mounted on linen, with rollers and varnished*, at 1*s*. 6*d*. each copy, or 1*s*. 10*d*. post free, or 15*s*. per dozen. The Almanac can be ordered through any Bookseller or Newsagent.

### AMUSEMENTS WITH PROFIT FOR ALL AGES.

**Rules.**—All answers must be addressed to the PRIZE EDITOR, THE HOSPITAL, 38, Old Jewry, Cheapside, London, E.C., not later than the *first* post on Thursday next. The full name and address must in all cases be given, but a *nom de plume* may be added for publication. Only one side of the paper must be written on.

### FOR MEN AND WOMEN.

### "THE HOSPITAL" WORK COMPETITION.

Our Work Competition closed on Saturday, the 7th, and this week we have the pleasant task of announcing the awards. Adjudication was not by any means easy in some departments, as all the competitors had evidently done their best, and the results were very satisfactory.

### Painting.

There were very few competitors for this prize, and though the specimens sent in were on the whole good, we should be glad to have more to choose from. In view of the limited number of competitors we have decided to award the following prizes:—

1st. Mrs. WARNER, Norton, Stockton-on-Tees; for a panel; subject, "Lilies;" one guinea

2nd. Miss GERTRUDE NIXON, Hadley, Barnet; painting on wood; subject, "Winter Scene"; half-a-guinea.

The remaining £1 11s. 6d. is transferred to the next department.

### Dolls and Pieces of Work, in which the competition is very good. The dolls showed considerab e variety in costume, and were for the most part both neatly and tastefully dressed. The miscellaneous work included all manner of cosy garments for invalids, old and young; and we beg to offer our special thanks to Ludwig, who sent a box of knitted gloves, socks, &c., for children, because, she said, "she was sure we should find a use for them." After some consideration, we have, however, decided to award the prizes as follows:—

1st. Miss A. C. GAUNT, Alexandra House, Glunlea Road, St. Leonards-on-Sea; "A patient's writing table for use in bed, filled; and Nurse DINSDALE, St. Bartholomew's Vicarage, Bethnal Green; "Baby doll with elaborately-worked robe,"

who receive one guinea each. A second prize of half a-guinea each has been awarded to the following:

Nurse HALL, Oldham Infirmary, Lancashire; doll dressed as surgical nurse.

"HONNIE," (Miss TURLEY), 47, Upper Berkeley Street, W.; doll dressed as St. Mary's Staff Nurse.

Mrs. W. B. SEALY, 14, Witham Place, Boston; doll "Mercy," dressed in exact facsimile of the costume worn by the "Blue School Girls."

Miss SHONE'S doll, "Norwegian peasant," is very highly commended.

We purpose keeping the two nurse dolls for the adornment of our office, 38, Old Jewry, E.C.; the others will be distributed with the remainder of the articles among the various Metropolitan hospitals.

### III. Scrap Books.

Most of these are unusually gocd, but we had no hesitation in awarding the prize of one guinea to

Master A. G. S. M'CULLOCH, 11, Highbury Quadrant, N., who is easily first in taste, neatness, and originality. The other competitors, however, had evidently taken great pains, and all deserve commendation.

Space forbids our giving the names of all the competitors in each division, but we thank them all very heartily for their contributions, and hope that they all, and many others, will take part in our next work competition, the particulars and conditions of which will shortly be announced. At present we will only state that one of its features will be a competition for dolls dressed as nurses, each competitor to send a complete set—sister, staff-nurse, and probationer-representing the hospital whose uniform is given.

### Acrostic Competition.

A PRIZE OF ONE GUINEA will be given to the competitor who gains the highest number of marks for best original acrostics during the ensuing quarter. All acrostics sent in for this competition will be credited according to merit, twelve marks being the highest score given for one acrostic.

A PRIZE of HALF-A-GUINEA to be given to the competitor who gains the highest score for solution of acrostics set, including the one inserted on November 26th. One mark only will be given to the competitors who solve all the lights of each acrostic.

These competitions to alternate weekly.

*Next week*, therefore, credit will be given for *original acrostic No. 4.*

(*Continued in column* 3.)

### THE THEATRES.

THE holidays have caused theatres to be the chief topic of conversation during the week and so many new pieces have been put upon the stage that we cannot attempt to deal with all, though we mentioned a great number in our last issue. Pantomime has of course been to the fore, and we intend to devote this article to a description of the attempt which has been made at Covent Garden, not only to reestablish pantomime there, but to revive to some extent the old style of pantomime. Before doing so, we cannot refrain from alluding to the calamity which led to the destruction of the Grand Theatre at Islington. Fortunately the performance was over, and the theatre closed for the night, so that the fire was not accompanied by that terrible loss of life which marked the destruction of the Exeter Theatre earlier in the year. Gas was apparently the cause of the fire, which spread very rapidly in spite of the fact that the theatre was specially constructed of fireproof material. The manager (Mr. Wilmot) and his family were only rescued with difficulty; but though saved, he and his burnt-out company are entitled to full sympathy for their grievous loss on the eve of what promised to be a most prosperous career. After months of the most careful preparation and toil, all plans have been upset, and the means of livelihood snatched away from 250 people in one fell moment.

To return to Covent Garden. Messrs. Freeman Thomas and W. T. Purkiss have spared no pains to provide a pantomime which should suit the tastes of all playgoers. The story of "Jack and the Beanstalk" has been followed with a trifle more exactness than is generally vouchsafed the old fairy tales, but the libretto is weak, and there is nothing taking in it, so that success will depend principally upon the performers and the "side shows." No less than ten scenes before the transformation scene are necessary to tell the story, and this spreads over four hours, without any rest or intermission whatever. This has become the established custom of the day, but it is certainly very trying to have to sit through so long a time, when there are many parts which might well be curtailed, and surely some interval might be given. Of these scenes some are very pretty and no expense has been spared in mounting the piece efficiently. The Village of Cowslipdale, with its smithy and working windmills, and Butterfly Land are amongst the best. Mr. George Conquest jun., is a past master in the art of playing the Giant, and he is most effective as Fee-Fi-Fo-Fum; his get up being so remarkably good, that it would be difficult to say exactly where the real man ends and where the "additions" begin. Mr. Sam Wilkinson acts the part of the Giant's Wife with spirit, and plays well with the Widow Simpson, Jack's mother (Mr. Frank Wood). Miss Fannie Leslie appears as Jack, and acts, sings, and dances with much spirit. The usual overgrown schoolboy appears, Billy Loblolly (Mr. Tom Squire), and he gives some fair comic business. To Miss Mario falls the duty of playing the Prince Amoroso, and the Misses Mayland and Hina Norince appear as the two indispensable heroines.

In Scene II. a very pretty and effective ballet is given, contrasting the costumes and dances of 1837 and 1887, outrivalled by the Butterfly Ballet in Scene IV., in which Signora Sozo scores a great success as the Queen. One of the best parts of the show is the drilling of the small boys in Scene II., who, dressed as French soldiers of the line, march on to the strains of the Boulanger song "Revenant de la Revue," and go through various bayonet exercises and drill, for which they receive rapturous applause. After the slaughter of the giant an occasion is formed for a long procession, the Seven Champions and their numerous followers marching across the stage. As we have said above, however, one of the best parts of the pantomime will be found to be the variety shows. M Cascabel, a Frenchman, is very clever in his change of dress on the stage, which is done almost instantaneously. Signor Carlo Huberti is clever in his imitation of singing birds, and in Scene VII. Little Sandy leads a merry rout by the cooks; but best of all are the Musical Jees and the two clever black cooks, Messrs Griffin and Ardell, in their scene, "Fun on the Quiet." Much good tumbling is thus introduced into the body of the pantomime, which concludes with a grand transformation scene. A harlequinade follows, but does not begin till eleven o'clock.

### FOR MEN AND WOMEN.

(*Continued from column* 1.)

### Results of No. 3.—Original Double Acrostic.

Brownie (12), Pasteur (12), Mater (12) E.M.B. 12), A.M.E. (11), Padre (10), Gretta (10), Condorcet (8).

### II. Word and Literary Competition.

Commenced November 5th, 1887; ends January 28th, 1888.

Three prizes of one guinea, 15s. and 10s. 6d. will be given each quarter to the three competitors who obtain the highest number of marks: (1) by making the largest number of words derived from the word or phrase set for anatomical dissection; or (2) for the best answers to (6); or (3) by (1) and 2) combined.

(A) THE WORD COMPETITION.

We shall give the newspapers for dissection during this quarter, that for this week being

"THE FORTNIGHTLY"

OBSERVE.—Proper names, abbreviations, foreign words, words of less than four letters and repetitions are barred. Nuttall's *Standard* dictionary only to be used.

### Results of Ninth Week.

THE QUEEN.—Brisbane (8), Gretta (8), Reldas (6), Condorcet (7), Lauriston (7), Ellice (6), Skye (7, Brownie (6).

### THE CHILDREN'S CORNER.

PRIZES.

FOR MOST CORRECT ANSWERS TO PUZZLES.

This Commenced October 1st, 1887.
One Pound a Month.

A PRIZE OF THREE GUINEAS

to the Competitor who shall have sent in the largest number of correct or best answers during the twelve months.

### Puzzles—Second Week.

(No. 5. GEOGRAPHICAL CONUNDRUM.—What river of Scotland is often mentioned in Spain?

. . . . No. 6 SQUARE WORD.—Part of
. . . . your body. 2. Caused by a chill. 3.
. . . . Fruit. 4. Found in a school-room.
. . . .

No. 7. HISTORICAL ACROSTIC.—1. A battle celebrated by the poet Campbell. 2. A battle fought in the reign of Henry V. 3. One of the battles fought during the Wars of the Roses. 4. The battle after which Margaret of Anjou's son was slain. 5. Earl Strongbow's conquest. 6. A battle in which David, king of Scotland, was taken prisoner. 7. Massacre of the MacDonalds. 8. A battle of Henry VIII The initials will give the name of a celebrated battle.

No. 8. ENIGMA.—What is that which is above all human imperfections and yet shelters and protects the weakest, wickedest, as well as the wisest of mankind

### Results of Fifth Week Puzzles.

No. 17. ENIGMA —Fix, IX., X.

No. 18. DOUBLE ACROSTIC.

S ali C
A nge L
N iagar A
T U (Tulip)
A nonymou S

No. 19 FRENCH PUZZLE SENTENCE.—J'aime en silence (six lances).

No. 20. BIOGRAPHICAL CHARADE.—Bloomfield=Bloomfield.

*All right*—Military Cadet. *Three right*—Judy, A.C.K., Pepita, Tim, Excelsior, Happy Eliza, Spider, Jumps, Scrooge. *Two right*—Plummy, Fluff, Skye, Ossian, A.G S., McCulloch, Granfer. *One right*—Gem, Fern.

### Results of Third Monthly Competition.

This month we again have a tie between Scrooge (Miss Constance L. Mills, 7, Charles-street, Pendleton), aged 13, and Tim (Master Cecil Anstey, 25, Dornton-road, Balham), aged 11, who have each succeeded in gaining the highest score (19 marks). We therefore divide the prize of £1 between them.

**Notice to all Correspondents.**—N.B.—All letters referring to this page, which do not arrive at 38, Old Jewry, Cheapside, London, E.C., by the *first post on Thursdays*, and are not addressed PRIZE EDITOR, will in future be disqualified and disregarded.

No one will be permitted to win the Monthly or Quarterly Prizes twice in any one year, the annual prizes being offered especially to prevent this.

